# l-Arginine as Bio-Based Curing Agent for Epoxy Resins: Temperature-Dependence of Mechanical Properties

**DOI:** 10.3390/polym14214696

**Published:** 2022-11-03

**Authors:** Florian Rothenhäusler, Holger Ruckdaeschel

**Affiliations:** 1 Department of Polymer Engineering, University of Bayreuth, Universitätsstraße 30, 95447 Bayreuth, Germany; 2 Neue Materialien Bayreuth GmbH, Gottlieb-Keim-Straße 60, 95448 Bayreuth, Germany

**Keywords:** l-arginine, sustainability, epoxy resin, DMA, tensile strength, fracture toughness, amino acid

## Abstract

The precise characterization of new bio-based thermosets is imperative for the correct assessment of their potential as matrix material in fiber-reinforced polymer composites. Therefore, the mechanical properties of diglycidyl ether of bisphenol a (DGEBA) cured with l-arginine were investigated to determine whether the bio-based thermoset possesses the required mechanical properties for application as a matrix material. The cured thermoset is called Argopox. The mixture of amino acid and epoxy resin was prepared via three-roll milling and cured in the presence of an urea-based accelerator. The tensile, compression, flexural and toughness properties of Argopox were characterized at T=−40 ${}^{\circ }C$, 22 ${}^{\circ }C$ and 80 ${}^{\circ }C$ to determine the temperature-dependence of the thermoset’s mechanical properties in its service temperature range. The glass transition temperature $T_{g}$ was analyzed via dynamic mechanical analysis (DMA) and is approximately 119 ${}^{\circ }C$. The tensile, compression and flexural strength at 22 ${}^{\circ }C$ are about 56 MPa, 98 MPa and 85 MPa, respectively. The critical stress intensity factor $K_{\mathrm{IC}}$ and fracture energy $G_{\mathrm{IC}}$ at 22 ${}^{\circ }C$ are roughly 1.1 MPam^0.5^ and 510 Jm^−^, respectively. Consequently, Argopox possesses mechanical properties that reach performance levels similar to that of materials which are already used as matrix for fiber reinforced composites.

## 1. Introduction

Fiber reinforced polymer composites are commonly used in industries like sports, automotive and aerospace due to their high weight-specific strength and modulus [1]. Here, the matrix properties dictate the composite’s maximum service temperature, its resistance against chemicals and corrosion as well as its mechanical properties perpendicular to fiber direction, like tensile strength, compression strength and inter-laminar strength [2]. Epoxy resins are important matrix systems for fiber composites due to their high mechanical properties, high $T_{g}$ and low viscosity during fiber impregnation [3]. However, standard curing agents for epoxy resins, such as amines, anhydrides and phenolic compounds, are harmful to health [4,5,6,7]. In addition, these curing agents are based on petroleum and are therefore not sustainable.

**Figure 1:**
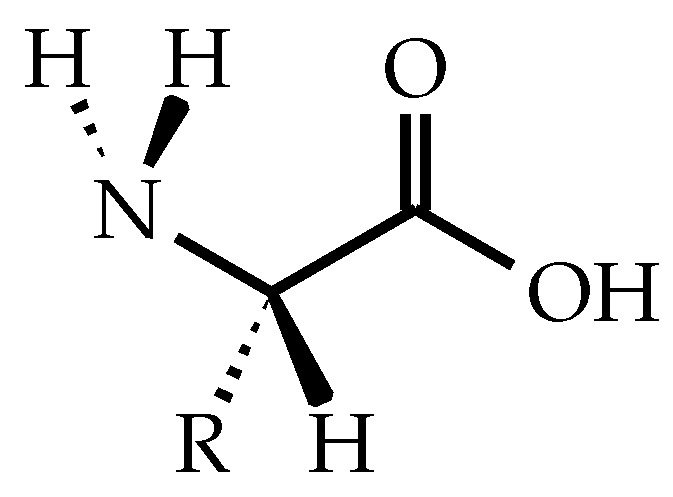
Structure of an l-amino acid that possesses an α-amine group (-NH_2_), carboxylate group (-COOH) and a side chain (-R).

Unlike petroleum-based amine hardeners, amino acids are nontoxic, biobased, and biodegradable components [8,9]. As their name suggests, amino acids contain amine (-NH_2_) and carboxylate (-COOH) functional groups, along a side chain (-R) that is different for each amino acid (see Figure 1) [10]. Since amino acids have the same amine groups as their petroleum-based counterparts, they are an environmentally friendly alternative as curing agents for epoxy resins.

**Figure 2:**
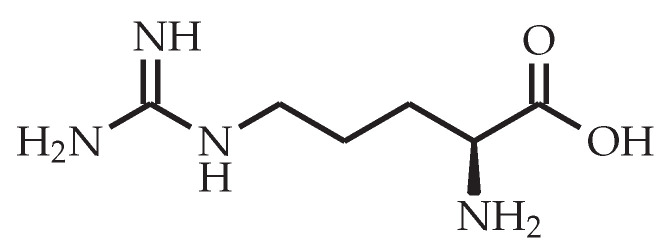
Chemical structure of l-arginine.

Previous investigations on epoxy resins cured with amino acids focused mainly on the reaction kinetics and $T_{g}$ of diglycidyl ether of bisphenol A (DGEBA) cured with l-tryptophan [11,12,13,14,15,16]. Rothenhäusler et al. [17] investigated the $T_{g}$ and viscosity of DGEBA cured with l-arginine (see Figure 2) as well as its latency at *T* = 22 ${}^{\circ }C$ and *T* = −18 ${}^{\circ }C$. They found that the thermoset possesses a $T_{g}$ of about 100 ${}^{\circ }C$, while its viscosity and latency are suitable for prepreg production.

Shibata et al. [18] investigated, among other things, the tensile properties of a bio-based epoxy resin derived from sorbitol polyglycidyl ether cured either with l-arginine, l-cysteine or l-lysine. The tensile strength of the thermosets is 8.5 MPa, 10 MPa and 26.7 MPa, respectively. However, the dimensions of tensile specimens were only 45 mm by 7 mm by 0.5 mm which are considerably smaller than the standardized 1B dog-bone specimens ( 150 mm by 10 mm by 4 mm) according to DIN EN ISO 527-2. Thus, there is a lack of information about the mechanical properties of amino acid cured epoxy resins. As the tensile strength and fracture toughness were not investigated sufficiently, the question remains unanswered whether epoxy resins cured with amino acids can be used as matrix for fiber reinforced composites.

Therefore, the objective of this investigation is to characterize the thermo-mechanical behavior, tensile, compression, flexural and fracture toughness properties at *T* = −40 ${}^{\circ }C$, 22 ${}^{\circ }C$ and 80 ${}^{\circ }C$ of DGEBA cured with l-arginine in the presence of an urea-based accelerator. This represents the first mechanical characterization of an epoxy resin cured with an amino acid with standardized specimens at different temperatures. In this study, l-arginine is used due to its high number of active hydrogen atoms, as well as its low price and good availability. The goal is to check whether the thermoset possesses the required mechanical properties for application as matrix material for fiber reinforced polymers.

## 2. Materials and Methods

### 2.1. Materials

D.E.R. 331 with an epoxide equivalent weight of 187 g mol^−1^ was purchased from Blue Cube Assets GmbH & Co. KG, Olin Epoxy (Stade, Germany). l-arginine with a purity of 98.9% was bought from Buxtrade GmbH (Buxtehude, Germany). The urea-based accelerator DYHARD®UR500 was bought from Alzchem Group AG (Trostberg, Germany).

### 2.2. Resin Formulation

The preparation of the mixture of DGEBA and l-arginine follows the procedure already applied and described in [17]. One weight percentage of DYHARD®UR500 (see Table 1) was added before mixing in a dual asymmetric centrifuge speed mixer by Hauschild Engineering (Hamm, Germany) at 3000 min^−1^ for 120 s. Afterwards, the mixture was degassed for 15 min at 10 mbar to ensure the elimination of entrapped air prior to curing. For the sake of simplicity, the cured thermoset is referred to as Argopox during this investigation.

### 2.3. Curing Cycle and Sample Preparation

The amino acid epoxy mixture was poured into aluminum molds that were pre-heated at 90 ${}^{\circ }C$. Afterwards, the material system was cured for 2 h at 120 ${}^{\circ }C$ and 2 h at 170 ${}^{\circ }C$ in an Memmert ULE 400 convection oven by Memmert GmbH + Co. KG (Schwabach, Germany). The maximum temperature of 170 ${}^{\circ }C$ was chosen to avoid the degradation of the amino acid. To prevent the build-up of internal stresses, the molds were cooled down to room temperature over 4 h. The specimens were prepared from the cured plates according to the ISO standards for each test method with a Mutronic DIADISC5200 diamond plate saw and CNC milled by a Mutronic Diadrive 2000 by MUTRONIC Präzisionsgerätebau GmbH & Co. KG (Rieden am Forggensee, Germany).

### 2.4. Characterization Methods

#### 2.4.1. Dynamic Mechanical Analysis

Thermo-elastic properties of the thermoset, such as storage modulus $E^{\prime }$, loss modulus $E^{\prime \prime }$ and loss factor tanδ, were investigated via dynamic mechanical analysis on a Gabo Eplexor 500 N (Gabo Qualimeter Testanlagen GmbH Ahlden, Germany) in tension mode. The specimens with dimensions 50 mm by 10 mm by 2 mm were measured between *T* = −120 ${}^{\circ }C$ and 200 ${}^{\circ }C$ with a constant heating rate of 3 K min^−1^. The tensile force amplitude was set to 60 N with a frequency of 1 Hz. Here, the glass transition temperature $T_{g}$ was chosen as the temperature at which the loss factor tanδ has its peak value. The cross-link density of the thermoset $\nu_{C}$ in the rubbery state was calculated as

(1) $$\nu_{C}=\frac{E^{\prime }}{3RT}\;,$$

with the storage modulus $E^{\prime }$ at *T* = $T_{g}$ + 50 K and the universal gas constant *R* = 8.314 J mol^−1^ K^−1^ [19]. Three specimens were tested to ensure adequate reproducibility of the results.

#### 2.4.2. Tensile and Compression Tests

For the tensile tests, 1B dog-bone specimens with dimensions 150 mm by 10 mm by 4 mm were tested with a cross-head speed of 5 mm min^−1^ according to DIN EN ISO 527-2. In order to determine the influence of temperature on tensile modulus, tensile strength and strain at failure, 8 specimens were tested at *T* = −40 ${}^{\circ }C$, 22 ${}^{\circ }C$ and 80 ${}^{\circ }C$, respectively. The thermoset’s compression strength was investigated according to EN ISO 604 on specimens with dimensions 10 mm by 10 mm by 4 mm. Ten specimens were tested with a cross-head speed of 5 mm min^−1^ at *T* = −40 ${}^{\circ }C$, 22 ${}^{\circ }C$ and 80 ${}^{\circ }C$, respectively. Tensile and compression tests were carried out on a ZwickRoell Z020 universal testing machine by ZwickRoell GmbH & Co. KG (Ulm, Germany) with a load cell with a capacity of 20 kN.

#### 2.4.3. Three-Point Bending

For the three-point bending tests, six specimens with dimensions 80 mm by 10 mm by 4 mm were tested with a cross-head speed of 2 mm min^−1^ according to ISO 178. Three-point bending tests were carried out on a ZwickRoell Z020 universal testing machine by ZwickRoell GmbH & Co. KG (Ulm, Germany) with a load cell with a capacity of 20 kN.

#### 2.4.4. Fracture Toughness

The critical stress intensity factor in mode I $K_{\mathrm{IC}}$ and fracture energy $G_{\mathrm{IC}}$ were determined according to ISO 13586 on a ZWICK Z020 by ZwickRoell GmbH & Co. KG (Ulm, Germany) equipped with a load cell with a capacity of 20 kN. The fracture energy is calculated from $K_{\mathrm{IC}}$ via:

(2) $$G_{\mathrm{IC}}=\frac{{K_{\mathrm{IC}}}^{2}}{E}\;\left(1-\nu^{2}\right)\;,$$

with Young’s modulus *E* taken from tensile tests and Poisson’s ratio ν, which is about 0.35 in the glassy state of the thermoset [20]. To determine the influence of temperature, nine specimens were tested at *T* = −40 ${}^{\circ }C$, 22 ${}^{\circ }C$ and 80 ${}^{\circ }C$, respectively.

The diameter of the plastic zone $d_{p}$ was calculated under the assumption of a plain strain behavior according to Irwin [21] via:

(3) $$d_{p}=\frac{1}{3\pi }{\left(\frac{K_{\mathrm{IC}}}{\sigma_{y}}\right)}^{2}\;,$$

with compression yield strength $\sigma_{y}$. The plastic zone is a region around the crack tip where the material deforms plastically during crack propagation and helps to compare materials regarding their resistance to crack propagation.

#### 2.4.5. Scanning Electron Microscopy

The fracture surfaces of compact tension specimens were characterized with a Zeiss Gemini 1530 Scanning Electron Microscope by Carl Zeiss AG (Oberkochen, Germany). The acceleration voltage used was 3 kV and the surfaces were platinum-sputtered with a thickness of about 5 nm.

## 3. Results and Discussion

### 3.1. Dynamic Mechanical Analysis

**Figure 3:**
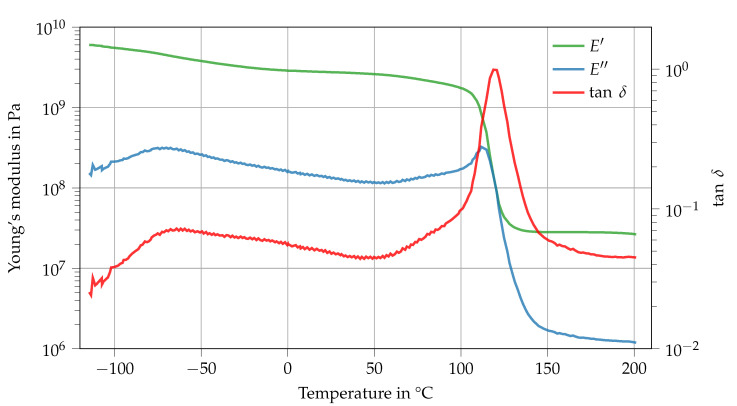
Dynamic mechanical analysis of Argopox between *T* = −120 ${}^{\circ }C$ and 200 ${}^{\circ }C$.

The storage modulus $E^{\prime }$ drops from 6 GPa at T= −120 ${}^{\circ }C$ to about 2.7 GPa at room temperature (see Figure 3). Here, the loss modulus $E^{\prime \prime }$ and subsequently the loss factor tanδ peak at around T= −75 ${}^{\circ }C$. This β-relaxation results from the increased distance between network segments which facilitates the rotation of individual molecular groups in a network segment. Garcia et al. [22] concluded from their investigation of DGEBA cured with different amines that the β-relaxation results from the hydroxy ether and diphenyl propane groups of DGEBA [23,24]. The $T_{g}$, meaning the temperature at which the loss factor tanδ has its peak value, of Argopox is approximately 119 ${}^{\circ }C$, whereas the $T_{g}$ of DGEBA cured with dicyandiamide is considerably higher at around 120 ${}^{\circ }C$ to 160 ${}^{\circ }C$ [25,26,27]. It is likely that the lower $T_{g}$ of Argopox compared to DGEBA cured with dicyandiamide stems from the aliphatic side chain of l-arginine which contains three carbon atoms between its α-amine group and the next functional group. The carbon chain is highly flexible which facilitates rearrangements of network segments and thus lowers $T_{g}$ [17]. During the glass transition, the storage modulus $E^{\prime }$ drops down to about 28 MPa. Consequently, the cross-link density $\nu_{C}$ is about 2540 mol m^−3^ which is comparable to epoxy resin cured with dicyandiamide [28].

### 3.2. Tensile Tests

**Figure 4:**
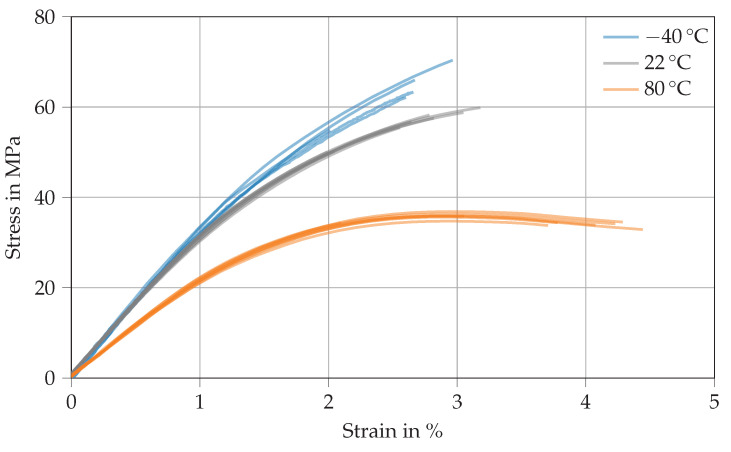
Stress–strain curves derived from tensile tests of Argopox at *T* = −40 ${}^{\circ }C$ (blue), 22 ${}^{\circ }C$ (gray), and 80 ${}^{\circ }C$ (orange).

Figure 4 shows various stress–strain curves derived from the tensile testing of Argopox at *T* = −40 ${}^{\circ }C$ (blue), 22 ${}^{\circ }C$ (gray), and 80 ${}^{\circ }C$ (orange). The thermoset’s tensile modulus decreases with increasing temperature from 3.4 GPa at −40 ${}^{\circ }C$ to 3.2 GPa at 22 ${}^{\circ }C$ and from there down to 2.2 GPa at 80 ${}^{\circ }C$ (see Table A1). The decrease of tensile modulus results from the increased distance between network segments and the weakening of secondary bonds between network segments. Similarly, the increased network segment distance results in a lower tensile strength which drops from 63.5 MPa at −40 ${}^{\circ }C$ to 35.7 MPa at 80 ${}^{\circ }C$. The increase of tensile strain at failure with increasing temperature might result from locally confined plastic deformation that might occur at 80 ${}^{\circ }C$ which is already close to the thermoset’s $T_{g}$ (see Table 2). Therefore, there is a slight transition from brittle failure at room temperature to minimal plastic flow at 80 ${}^{\circ }C$. Similarly to the $T_{g}$, the tensile strength of Argopox is lower than that of DGEBA cured with dicyandiamide [29,30]. It is likely that the reduced strength is the result of the long aliphatic side chain of l-arginine. Another problem might be the formation of water which could happen during the peptide reaction between amino acids or during the esterification of l-arginine’s carboxylate group with a hydroxyl group of DGEBA [31,32,33].

### 3.3. Compression Tests

**Figure 5:**
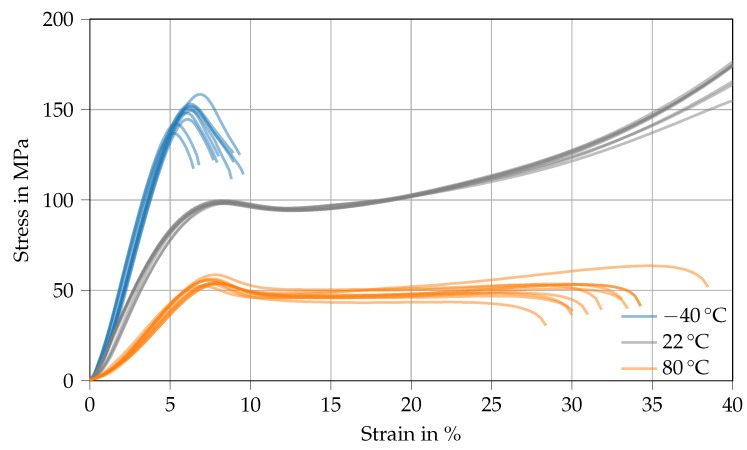
Stress–strain curves derived from compression strength tests of Argopox at *T* = −40 ${}^{\circ }C$ (blue), 22 ${}^{\circ }C$ (gray), and 80 ${}^{\circ }C$ (orange).

Figure 5 shows various stress–strain curves derived from compression testing of Argopox at *T* = −40 ${}^{\circ }C$ (blue), 22 ${}^{\circ }C$ (gray) and 80 ${}^{\circ }C$ (orange). The thermoset’s compression yield strength decreases with increasing temperature from 149.1 MPa at −40 ${}^{\circ }C$ to 98.4 MPa at 22 ${}^{\circ }C$ and from there down to 54.6 MPa at 80 ${}^{\circ }C$. Therefore, the compression yield strength of Argopox at room temperature (98.4 MPa) is comparable to that of DGEBA cured with dicyandiamide in the presence of DYHARD®UR500 (113.7 MPa) [30]. Once again, the decrease of compression strength results from the increased distance between network segments and the weakening of secondary bonds between network segments. However, the compression yield strain remains about the same for the tested temperatures and is approximately 6 to 8.5 (see Table A1). Interestingly, the compression strain at which the thermoset fails is largest at 22 ${}^{\circ }C$, whereas the brittle behavior at −40 ${}^{\circ }C$ and the low strength at 80 ${}^{\circ }C$ facilitate failure at a smaller strain.

### 3.4. Three-Point Bending

**Figure 6:**
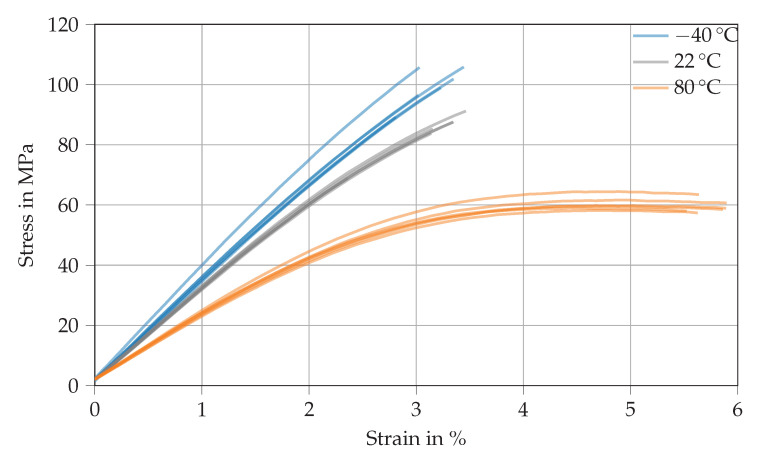
Stress–strain curves derived from three-point bending tests of Argopox at *T* = −40 ${}^{\circ }C$ (blue), 22 ${}^{\circ }C$ (gray), and 80 ${}^{\circ }C$ (orange).

Figure 6 shows various stress–strain curves derived from three-point bending of Argopox at *T* = −40 ${}^{\circ }C$ (blue), 22 ${}^{\circ }C$ (gray) and 80 ${}^{\circ }C$ (orange). Similar to the results of tensile and compression testing, the thermoset’s flexural modulus decreases with increasing temperature from 3.3 GPa at −40 ${}^{\circ }C$ to 2.2 GPa at 80 ${}^{\circ }C$. Accordingly, flexural strength decreases and flexural strain at failure increases with increasing temperature (see Table A1). Usually, specimen failure is initiated at the side where tensile stresses act as the thermoset’s tensile strength is considerably lower than its compression yield strength. As with the tensile and compression yield strength, the flexural strength of Argopox at room temperature ( 84.7 MPa) is lower than that of DGEBA cured with petroleum-based amines ( 95 MPa to 123 MPa) [22].

### 3.5. Fracture Toughness

Table A1 shows the critical stress intensity factor in mode I $K_{\mathrm{IC}}$ and fracture energy $G_{\mathrm{IC}}$ of Argopox at *T* = −40 ${}^{\circ }C$, 22 ${}^{\circ }C$ and 80 ${}^{\circ }C$. Here, the resistance against critical crack growth $K_{\mathrm{IC}}$ is virtually independent of temperature and about 1.1 MPa m^0.5^. Contrary to that, the fracture energy $G_{\mathrm{IC}}$ that is necessary for crack growth increases from 419 J m^−2^ at −40 ${}^{\circ }C$ to 805 J m^−2^ at 80 ${}^{\circ }C$. At the same time, the diameter of the plastic zone $d_{p}$ increases from 5.08 μm to 46.09 μm. As $K_{\mathrm{IC}}$ is independent from temperature, it is likely that the main toughening mechanisms change with a change in temperature.

**Figure 7:**
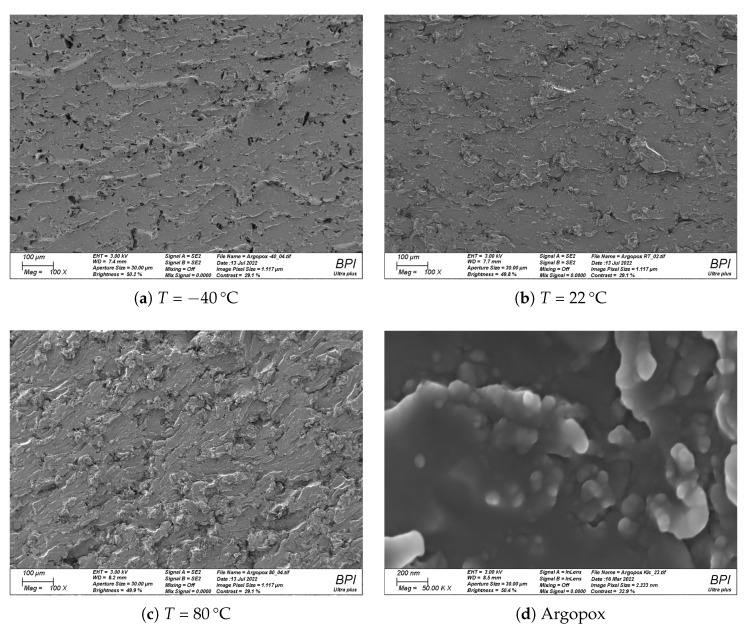
Fracture surfaces of compact tension specimens made from Argopox after testing at *T* = −40 ${}^{\circ }C$ (**a**), 22 ${}^{\circ }C$ (**b**), and 80 ${}^{\circ }C$ (**c**). Scanning electron microscopy image of cured Argopox at 50,000 × magnification showing the nanometer-sized l-arginine particles (**d**) [17].

Normally, $K_{\mathrm{IC}}$ of unfilled epoxides are in the range of 0.6 to 0.7 MPa m^0.5^ [30,34,35]. However, the nanometer-sized particles of l-arginine that are dispersed throughout the thermoset act as toughening agent (see Figure 7d). Since the amino acid acts as curing agent and toughening agent at the same time, amino acid cured epoxy resins, like Argopox, are intrinsically toughened by their hardener. It can be assumed that the amino acid particles do not decrease the strength of Argopox significantly as the particles are chemically identical to the curing agent in the thermoset and the particles are well bonded via the amine-epoxy reaction to the surrounding matrix. Here, the toughening effect of l-arginine particles in epoxy resin is less than that of carboxy-terminated butadiene–acrylonitrile rubbers [36] and more comparable to that of core-shell particles [37]. However, the results require further investigation to understand the micromechanics of the toughening modification of arginine particles which is beyond the scope of this study.

At −40 ${}^{\circ }C$, the fracture surface is rough and shows voids (see Figure 7a), whereas the fracture surface of the specimen tested at 22 ${}^{\circ }C$ shows less voids and a rougher surface (see Figure 7b). This trend continues, with the fracture surface at 80 ${}^{\circ }C$ showing an even rougher surface compared to at 22 ${}^{\circ }C$ (see Figure 7c). It is likely that particles get pulled out and local plastic deformation is minimal at low temperatures, whereas the tendency for local plastic deformation at the crack tip increases with increased temperature. Furthermore, it is probable that the particles induce crack pinning and crack deflection.

## 4. Conclusions

This study focused on the temperature-dependence of the tensile, compression, flexural and fracture toughness properties of DGEBA cured with l-arginine in the presence of an urea-based accelerator. This represents the first mechanical characterization of an amino acid cured epoxy resin using standardized specimens. As expected from a viscoelastic material, an increase in temperature leads to a decrease of modulus and strength, while the fracture strain increases. The tensile, compression and flexural strength at 22 ${}^{\circ }C$ are about 56 MPa, 98 MPa and 85 MPa respectively and thus comparable to but still slightly lower than those of DGEBA cured with dicyandiamide or aliphatic amines. The $T_{g}$ of Argopox is approximately 119 ${}^{\circ }C$, whereas $T_{g}$ of DGEBA cured with dicyandiamide is considerably higher at around 120 ${}^{\circ }C$ to 160 ${}^{\circ }C$. It is likely that the lower strength and $T_{g}$ of Argopox compared to DGEBA cured with dicyandiamide stems from the aliphatic side chain of l-arginine which contains three carbon atoms between its α-amine group and the next functional group. The carbon chain is highly flexible which facilitates rearrangements of network segments and thus lowers $T_{g}$. The critical stress intensity factor $K_{\mathrm{IC}}$ is almost independent of temperature and roughly 1.1 MPa m^0.5^, whereas $G_{\mathrm{IC}}$ increases significantly with increased temperature. In principle, the nanometer-sized l-arginine particles act as toughening agent. As there are 22 proteinogenic amino acids, it would be interesting to see the effect of different amino acids on the mechanical properties of the resulting thermoset. Since Argopox possesses a low viscosity for prepreg production, as well as sufficient latency [17] and mechanical properties that reach performance levels similar to that of materials which are already used as matrix materials over a wide temperature range, it is suitable as matrix for sustainable fiber reinforced composites.

## Figures and Tables

**Table 1:** Composition of Argopox.

Component	Argopox
D.E.R. 331	87.4 wt.%
l-arginine	11.6 wt.%
DYHARD®UR500	1 wt.%

**Table 2:** Key data of Argopox derived from dynamic mechanical analysis (average ± standard deviation).

Physical Quantity	Value
$T_{g}$ (max. tanδ) in ${}^{\circ }C$	119.1 ± 0.45
Cross-link density $\nu_{C}$ in mol $m^{-3}$	2540
$E^{\prime }$ at T= −120 ${}^{\circ }C$ in GPa	5.9 ± 0.1
$E^{\prime }$ at T= 22 ${}^{\circ }C$ in GPa	2.7 ± 0.1
$E^{\prime }$ at T= 80 ${}^{\circ }C$ in GPa	2.1 ± 0.1

## Appendix A

**Table A1:** Overview of tensile, compression, flexural, and fracture toughness properties of Argopox (average ± standard deviation) at T=−40 ${}^{\circ }C$, 22 ${}^{\circ }C$, and 80 ${}^{\circ }C$.

	*T* = −40 ${}^{\circ }$C	*T* = −22 ${}^{\circ }$C	*T* = −80 ${}^{\circ }$C
Tensile modulus in GPa	3.4 ± 0.1	3.2 ± 0.1	2.2 ± 0.1
Tensile strength in MPa	63.5 ± 5.1	56.0 ± 3.5	35.7 ± 0.7
Tensile strain in	2.59 ± 0.31	2.65 ± 0.41	3.77 ± 0.73
Compression yield strength in MPa	149.1 ± 5.6	98.4 ± 1.2	54.6 ± 1.65
Compression yield strain in	6.1 ± 0.43	8.51 ± 0.38	7.46 ± 0.37
Flexural modulus in GPa	3.3 ± 0.2	2.9 ± 0.1	2.2 ± 0.1
Flexural strength in MPa	100.5 ± 5.9	84.7 ± 6.9	60.6 ± 2.1
Flexural strain in	3.18 ± 0.22	3.17 ± 0.36	5.81 ± 0.14
$K_{\mathrm{IC}}$ in $M\mathrm{Pa}m^{0.5}$	1.05 ± 0.09	1.09 ± 0.14	1.14 ± 0.07
$G_{\mathrm{IC}}$ in J m^−2^	419 ± 47	510 ± 132	805 ± 113
$d_{p}$ in μm	5.1	13.1	46.1
